# Comparison of Slovak reference values for anthropometric parameters in children and adolescents with international growth standards: implications for the assessment of overweight and obesity

**DOI:** 10.3325/cmj.2018.59.313

**Published:** 2018-12

**Authors:** Valéria Regecová, Jana Hamade, Hana Janechová, Ľudmila Ševčíková

**Affiliations:** 1 Slovak Academy of Sciences, Center of Experimental Medicine, Institute of Normal and Pathological Physiology, Bratislava, Slovakia; 2 Department of Children and Youth Hygiene, Public Health Authority of the Slovak Republic, Bratislava, Slovakia; 3 Faculty of Medicine, Comenius University, Institute of Hygiene, Bratislava, Slovakia

## Abstract

**Aim:**

To compare the national reference percentile values for body height, weight, and body mass index (BMI) of children and adolescents in Slovakia with international standards and to analyze growth trends in this population.

**Methods:**

The study was designed as a repeated cross-sectional survey. Two nationwide anthropometric surveys (NAS) performed in 2001 and 2011 assessed body weight, height, and BMI of 38 692 children aged 7 to 18 years. Age- and sex-specifıc smoothed percentiles were generated with the lambda-mu-sigma method. Slovak standards were compared with World Health Organization (WHO) 2007 z-scores and International Obesity Task Force (IOTF) standards.

**Results:**

Medians of body height corresponded to the 75th-85th percentile of the WHO 2007 standards. The secular trend of height increase was attenuated, and the final body height did not change between NAS 2001 and NAS 2011. The cut-off BMI values for obesity, set at the 97th percentile for age <14 years, were higher across age ranges than WHO 2007 standards but lower than IOTF standards. Obesity prevalence, relatively low in 2001 (<3%), doubled during the following decade (*P* < 0.001), with the highest values (4.8%-7.6%) observed in children aged up to 13 years.

**Conclusion:**

NAS 2001 data were chosen as national growth standards, as these data were not influenced by the obesity rates increase in the period between the surveys. BMI cut-offs were lower than those in most European countries. Obesity proportions in prepubertal and pubertal boys might be overestimated when WHO 2007 cut-offs are used.

Child and adolescent growth and development are important indicators of nutritional status and health. To identify developmental deviations and impending health problems, it is necessary to have adequate standards and reference values. International and generally accepted standards, such as those set by the World Health Organization (WHO) Child Growth Standards (1), Centers for Disease Control and Prevention (CDC) 2000 (2), and International Obesity Task Force (IOTF) (3) were derived from cross-sectional surveys using representative samples. They enable the comparison of growth patterns of different populations or epidemiological data estimates of malnutrition, overweight, and obesity. While the WHO 2007 classification provides z-scores of body height, weight, and BMI for sex and age (1), IOTF approach bases age- and sex-specific BMI cut-off points for overweight and obesity in children on percentiles analogous to the adult BMI of 25 and 30 kg/m^2^, respectively (3). However, these do not consider growth rate and secular population trends, which is why there is a need for standards that would more objectively assess developmental trends and nutritional status at individual and population levels.

Slovakia had had a sustained tradition of growth studies of children long before WHO recommendations were made. The first nationwide anthropometric survey (NAS) of children and adolescents in former Czechoslovakia was conducted in 1951. Notable differences between Czech and Slovak populations of children and youths observed in the first representative studies have been gradually decreasing (4,5). In the meantime, several studies used growth data to assess the impact of social, environmental, and behavioral factors (6-8). After the dissolution of Czechoslovakia, transverse representative surveys in Slovakia have continued in 10-year intervals and served as a valid source of reference data for the population aged 0-18 years. They showed secular changes in child and adolescent anthropometric parameters (body height, weight, and circumferences) (9,10).

The latest NAS was carried out in 2011. Since NAS 2001 did not exclude outliers and references took no account of skewness of weight and BMI, we revised and recalculated these standards according to Cole (11). The aim of this study was to provide up-to-date national reference values for school-aged children and adolescents aged up to 18 years in Slovakia. Moreover, we compared our nation-specific references with other EU countries (12-15) and international standards (1,3), to analyze time trends and compare overweight and obesity prevalence. Our hypothesis was that the latest BMI percentile distribution of school-aged children and adolescents in Slovakia would be substantially modified by the increasing obesity prevalence in industrialized countries (16,17), and that Slovak-specific reference values for both sexes would differ systematically from the international standards across age ranges, as WHO 2007 standards were based on American population assessed 40 years ago, and IOTF standards assessment included only two European populations (British and Dutch) (3) and was based on different principles than WHO and Slovak standards assessments.

## Participants and methods

### Participants

The study was designed as a repeated cross-sectional comparative survey using data from two surveys, NAS 2001 and NAS 2011. Both surveys were coordinated by the Ministry of Health and Institute of Hygiene, Faculty of Medicine of the Comenius University in Bratislava and approved by the Ethics Committee of the Public Health Authority of the Slovak Republic in Bratislava, No. 4/2001 and No. 12/2011, and performed in accordance with the Declaration of Helsinki. The parents were fully informed about all study procedures and provided the informed consent.

The survey was conducted in elementary and secondary schools in the entire territory of Slovakia. Data were collected by experienced staff of 36 Regional Public Health Authorities from all Slovakian districts. This ensured a balanced sampling proportional to the population density in both rural and urban parts of Slovakia. The lists of primary and secondary school pupils were provided by the Ministry of Education, Science, Research and Sport of the Slovak Republic. Proportionate stratified random sampling according to the number of children and adolescents (separately boys and girls) in the respective age groups was performed by Regional Public Health Authorities. Since the region size and the number of children and adolescents in each region varied, the selection interval was chosen so that the number of participants from each region would amount to at least 1%-2% of the population of that age in the respective region. The NAS 2001 was conducted in September-October 2001 and the NAS 2011 in September-October 2011. Measurement procedures were based on the best recommended practices (18,19). Chronological age was calculated as the decimal age by subtracting the observation date from the birth date. The exclusion criteria were those defined by WHO and included diseases affecting growth (hormonal, metabolic, and genetic) and birth weight <1500 g (1). After anomalies and erroneous data (3%-5%) were removed, the final data set in NAS 2001 consisted of 20 596 participants and the final data set in NAS 2011 consisted of 18 096 participants. The population registry from the Statistical Office of Slovakia included 919 390 children and adolescents aged 7-18 years in 2001, and 692 973 in 2011. Therefore, our sample represented on average 2.3% of the entire population of interest in 2001 and 2.6% in 2011, ranging between 1.2%-3.1% depending on the age group.

### Methods

Body height was measured in barefoot participants standing against a wall (tape gauge mounted on the wall with zero at the pad) to the nearest 0.1 cm. Body weight was measured using a personal calibrated scale in participants in underwear, to the nearest 0.1 kg. BMI was calculated individually as the ratio of weight (kilograms) to height (meters) squared and rounded to 3 decimal places.

Reference values were determined using the LMS Chart Maker Pro software, version 2.54, developed by Pan and Cole (20), which fits smooth centiles to reference data using the lambda-mu-sigma (LMS) method (21). Z-scores, percentile values (3rd, 10th, 25th, 50th, 75th, 90th, and 97th), and curves were set at 0.5 years intervals. They were compared to the z-scores according to WHO 2007 and IOTF grades by LMSgrowth 2.77, a Microsoft Excel add-in to access growth references (22). The same program was used to identify 10-year time trends in anthropometric parameters measured by NAS 2001 and NAS 2011.

### Statistical analysis

The data are presented as mean values and standard deviations (SD) and percentile values by age and sex. Prevalence rates are expressed as percentages. Differences in mean body height and weight between NAS 2001 and NAS 2011 and between boys and girls were evaluated by ANOVA. The prevalence of overweight and obesity in NAS 2001 and NAS 2011 by sex and age groups was compared using χ^2^ test. The level of significance was set at *P* < 0.01. The statistical analysis was performed using Statgraphic Centurion (STATGRAPHICS® Centurion version XVI, StatPoint Technologies, Inc., The Plains, VA, USA).

## Results

### Descriptive statistics of basic anthropometric parameters

Distribution of participants included in NAS 2001 and NAS 2011 according to sex and age is presented in Table 1. Boys up to 12 years old grew yearly by an average of 5.3 cm. The growth velocity culminated between 12.5 and 14.5 years, with the height increment of 14.5 cm. Thereafter, the growth slowed down but continued until 18 years. Although growth acceleration in boys was more pronounced in NAS 2011 than in NAS 2001, the final mean height did not change. The median age at voice change in boys was 13 years (intersextile range 12-14 years). The median age at menarche was 12 years (intersextile range 11-13 years). The highest growth velocity in girls was observed from 10 to 12 years, with the increment of 12.8 cm. During this period, girls were significantly taller than boys according to NAS 2001, but not according to NAS 2011 (Table 2 and Table 3).

**Table 1:** Children and adolescents involved in nationwide anthropometric surveys (NAS) 2001 and NAS 2011 in Slovakia according to sex and age groups

	2001			2011		
Age (years)	boys	girls	all	boys	girls	all
**7**	879	874	1753	714	715	1429
**8**	988	946	1934	714	716	1430
**9**	817	825	1642	723	723	1446
**10**	746	774	1520	707	718	1425
**11**	781	850	1631	713	713	1426
**12**	816	852	1668	700	716	1416
**13**	872	859	1731	714	718	1432
**14**	524	464	988	707	706	1413
**15**	1361	1265	2626	838	818	1656
**16**	1148	1062	2210	854	837	1691
**17**	1161	1120	2281	857	827	1684
**18**	314	298	612	824	824	1648
**Total**	10 407	10 189	20 596	9065	9031	18 096

**Table 2:** Mean values of body height ± standard deviations (SD) in children and adolescents in the nationwide anthropometric survey (NAS) 2011 in Slovakia and their changes since NAS 2001

	Body height (mean ± SD)							
	Boys			Girls			Sex differences	
Age (years)	in 2011	changed since 2001		in 2011	changed since 2001		2001	2011
	cm	cm	*P*	cm	cm	*P*	*P*	*P*
**7**	127.5 ± 6.3	0.01	0.969	126.5 ± 6.3	0.19	0.684	<0.001	<0.001
**8**	132.9 ± 6.5	0.26	0.424	132.4 ± 6.2	0.41	0.240	<0.001	0.102
**9**	138.4 ± 6.7	-0.14	0.685	138.1 ± 7.2	1.07	<0.001	<0.001	0.381
**10**	144.2 ± 7.1	0.70	<0.001	144.3 ± 7.6	1.05	<0.001	0.490	0.915
**11**	150.3 ± 7.8	1.62	<0.001	150.6 ± 7.6	-0.12	0.601	<0.001	0.401
**12**	156.3 ± 8.6	1.17	0.007	157.1 ± 7.5	0.77	<0.001	<0.001	0.055
**13**	164.0 ± 8.8	1.15	<0.001	160.2 ± 7.0	-0.57	0.089	<0.001	<0.001
**14**	170.9 ± 8.6	2.26	<0.001	162.9 ± 6.7	-0.08	0.839	<0.001	<0.001
**15**	175.8 ± 7.6	0.75	0.020	164.6 ± 6.1	-0.81	0.003	<0.001	<0.001
**16**	177.6 ± 7.1	-0.52	0.097	164.8 ± 6.6	-1.02	0.030	<0.001	<0.001
**17**	178.4 ± 7.1	-0.70	0.020	165.2 ± 6.0	-0.60	0.003	<0.001	<0.001
**18**	179.3 ± 6.7	-0.25	0.542	165.4 ± 6.5	0.04	0.931	<0.001	<0.001

**Table 3:** Mean values of body weight ± standard deviations (SD) in children and adolescents in the nationwide anthropometric surveys (NAS) 2011 in Slovakia and their changes since NAS 2001

	Body weight (mean ± SD)							
	Boys			Girls			Sex differences	
Age (years)	in 2011	changed since 2001		in 2011	changed since 2001		2001	2011
	kg	kg	*P*	kg	kg	*P*	*P*	*P*
**7**	27.5 ± 6.4	1.5	<0.001	26.5 ± 6.1	1.2	<0.001	<0.001	<0.001
**8**	30.5 ± 7.2	1.6	<0.001	30.2 ± 6.9	1.9	<0.001	<0.001	0.040
**9**	34.4 ± 8.7	1.8	<0.001	34.0 ± 8.2	2.5	<0.001	<0.001	0.002
**10**	38.9 ± 10.0	3.1	<0.001	38.9 ± 9.6	3.6	<0.001	0.313	0.962
**11**	43.9 ± 11.0	4.4	<0.001	43.4 ± 10.5	2.4	<0.001	<0.001	0.461
**12**	49.3 ± 6.1	5.0	<0.001	48.7 ± 10.6	3.2	<0.001	<0.001	0.020
**13**	54.8 ± 13.0	4.2	<0.001	52.1 ± 10.4	1.8	<0.001	<0.001	<0.001
**14**	61.0 ± 12.7	4.8	<0.001	55.6 ± 11.0	3.4	<0.001	<0.001	<0.001
**15**	66.0 ± 12.9	3.9	<0.001	57.4 ± 10.2	1.9	<0.001	<0.001	<0.001
**16**	69.1 ± 12.6	3.0	<0.001	57.7 ± 10.5	0.8	0.080	<0.001	<0.001
**17**	72.1 ± 12.9	2.9	<0.001	58.2 ± 9.1	0.8	0.030	<0.001	<0.001
**18**	74.1 ± 12.2	3.9	<0.001	59.3 ± 10.1	1.8	0.011	<0.001	<0.001

Maximum weight gain in boys was delayed by 1 year after the growth spurt (+11.2 kg from 13-15 years). A similar delay was observed in girls, with an increment of 8-9 kg from 11-13 years (Table 3). The weight gain in girls considerably slowed down from the age of 13 to the age of 18 years (on average by 1.4 kg/y), but boys were still gaining 2.5 kg/y. Boys were heavier than girls (*P* < 0.001), except in the age group 10-11 years. In the 10-year period between the surveys, mean body weight significantly increased (*P* < 0.001) across the whole age range on average by 3.3 kg (1.5-5 kg) in boys and 2.1 kg (0.8-3.6 kg) in girls. In both surveys, after the age of 12 boys were taller (*P* < 0.001) and heavier than girls (*P* < 0.001). On the whole, significant increases in weight across the whole age range during the 2001-2011 decade were not proportional to height. Therefore, percentile reference values for anthropometric parameters were based on NAS 2001 data.

### Comparison of percentile values

**Figure 1:**
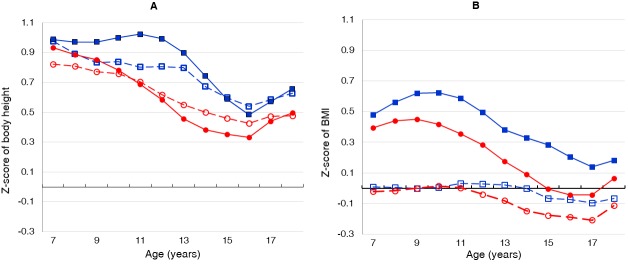
Z-scores according to World Health Organization (WHO) 2007 standards corresponding to medians of body height (**A**) and body mass index (BMI) values (**B**) in Slovak population computed from nationwide anthropometric surveys (NAS) 2001 and NAS 2011 data. Open square – boys 2001; open circle – girls 2001; filled square – boys 2011; filled circle – girls 2011.

Medians of height in our population were by 2.5-3.0 cm higher than WHO 2007 standards, corresponding approximately to z-score 0.6-1 or the 75th-85th percentile (Figure 1A), except for 14-17-year-old girls in 2001 (z-score from 0.33 to 0.475). Over the next 10 years, these values changed only slightly, by a maximum of 0.2 index units (IU) (Tables 4-9).

**Table 4:** Percentile values and z-scores of body height in boys derived from the nationwide anthropometric survey 2001 in Slovakia

z-score	-1.881	-1.282	-0.674	0	0.674	1.282	1.881
Percentile	3	10	25	50	75	90	97
Age (years)							
7.0	112.88	116.60	120.35	124.47	128.55	132.18	135.75
7.5	115.51	119.28	123.08	127.27	131.44	135.17	138.82
8.0	118.06	121.89	125.75	130.02	134.28	138.10	141.85
8.5	120.54	124.42	128.35	132.71	137.06	140.97	144.82
9.0	122.95	126.90	130.90	135.35	139.80	143.81	147.77
9.5	125.30	129.32	133.40	137.94	142.50	146.61	150.67
10.0	127.58	131.68	135.84	140.49	145.15	149.37	153.54
10.5	129.86	134.04	138.30	143.05	147.83	152.15	156.43
11.0	132.22	136.50	140.85	145.72	150.61	155.04	159.43
11.5	134.77	139.14	143.60	148.59	153.60	158.14	162.64
12.0	137.59	142.07	146.64	151.74	156.87	161.52	166.12
12.5	140.73	145.32	149.99	155.20	160.44	165.17	169.86
13.0	144.19	148.86	153.61	158.90	164.21	169.01	173.76
13.5	147.86	152.57	157.35	162.68	168.02	172.83	177.59
14.0	151.57	156.28	161.05	166.35	171.66	176.44	181.16
14.5	155.15	159.80	164.51	169.74	174.97	179.67	184.31
15.0	158.34	162.90	167.52	172.64	177.76	182.36	186.90
15.5	160.94	165.40	169.92	174.93	179.93	184.43	188.86
16.0	162.92	167.30	171.73	176.64	181.54	185.94	190.27
16.5	164.39	168.70	173.06	177.88	182.70	187.03	191.29
17.0	165.47	169.72	174.02	178.79	183.54	187.81	192.01
17.5	166.31	170.52	174.77	179.49	184.19	188.41	192.57
18.0	167.05	171.22	175.43	180.10	184.76	188.94	193.06
18.5	167.75	171.88	176.06	180.69	185.31	189.45	193.53

**Table 5:** Percentile values and z-score of body height in girls derived from the nationwide anthropometric survey 2001 in Slovakia

z-score	-1.881	-1.282	-0.674	0	0.674	1.282	1.881
Percentile	3	10	25	50	75	90	97
Age (years)							
7.0	112.47	115.95	119.50	123.45	127.42	131.00	134.55
7.5	114.90	118.47	122.10	126.15	130.22	133.90	137.55
8.0	117.31	120.96	124.69	128.84	133.03	136.81	140.57
8.5	119.73	123.48	127.30	131.57	135.87	139.77	143.63
9.0	122.20	126.06	129.99	134.38	138.81	142.81	146.79
9.5	124.81	128.77	132.82	137.33	141.88	146.00	150.08
10.0	127.58	131.65	135.81	140.45	145.11	149.33	153.51
10.5	130.52	134.70	138.96	143.70	148.46	152.76	157.02
11.0	133.61	137.87	142.19	147.01	151.83	156.18	160.48
11.5	136.73	141.04	145.40	150.25	155.09	159.45	163.75
12.0	139.78	144.09	148.45	153.28	158.09	162.41	166.66
12.5	142.64	146.92	151.24	156.01	160.75	165.00	169.18
13.0	145.24	149.46	153.71	158.40	163.05	167.21	171.29
13.5	147.52	151.67	155.84	160.43	164.98	169.05	173.03
14.0	149.44	153.52	157.61	162.11	166.56	170.53	174.42
14.5	151.00	155.01	159.03	163.44	167.81	171.71	175.51
15.0	152.20	156.16	160.12	164.47	168.77	172.60	176.34
15.5	153.08	156.99	160.91	165.21	169.46	173.24	176.94
16.0	153.66	157.55	161.43	165.70	169.92	173.67	177.33
16.5	154.01	157.88	161.75	166.00	170.19	173.92	177.56
17.0	154.20	158.05	161.92	166.15	170.33	174.05	177.69
17.5	154.28	158.13	161.99	166.22	170.40	174.11	177.74
18.0	154.31	158.17	162.02	166.25	170.42	174.14	177.77
18.5	154.33	158.18	162.04	166.26	170.44	174.15	177.78

**Table 6:** Percentile values and z-score of body weight in boys derived from the nationwide anthropometric survey 2001 in Slovakia

z-score	-1.881	-1.282	-0.674	0	0.674	1.282	1.881
Percentile	3	10	25	50	75	90	97
Age (years)							
7.0	18.01	19.50	21.28	23.69	26.71	30.19	34.66
7.5	18.85	20.42	22.32	24.90	28.17	31.96	36.87
8.0	19.79	21.47	23.50	26.28	29.81	33.96	39.39
8.5	20.80	22.59	24.77	27.76	31.59	36.13	42.16
9.0	21.86	23.77	26.09	29.30	33.46	38.41	45.08
9.5	22.91	24.95	27.43	30.88	35.36	40.74	48.05
10.0	23.95	26.12	28.77	32.45	37.26	43.07	50.98
10.5	25.01	27.32	30.14	34.08	39.23	45.44	53.93
11.0	26.13	28.60	31.62	35.83	41.33	47.96	56.97
11.5	27.37	30.02	33.27	37.78	43.65	50.68	60.13
12.0	28.77	31.65	35.16	40.01	46.27	53.67	63.47
12.5	30.40	33.54	37.35	42.57	49.22	56.96	66.97
13.0	32.33	35.75	39.88	45.48	52.50	60.52	70.62
13.5	34.55	38.27	42.71	48.66	56.01	64.20	74.29
14.0	37.06	41.04	45.76	51.99	59.57	67.87	77.85
14.5	39.80	43.98	48.89	55.31	63.03	71.37	81.25
15.0	42.57	46.86	51.86	58.37	66.13	74.45	84.24
15.5	45.16	49.47	54.48	60.99	68.72	76.99	86.68
16.0	47.45	51.73	56.71	63.16	70.81	79.01	88.63
16.5	49.41	53.65	58.57	64.94	72.52	80.64	90.20
17.0	51.10	55.28	60.14	66.44	73.94	81.99	91.50
17.5	52.60	56.73	61.54	67.77	75.19	83.17	92.63
18.0	54.03	58.11	62.85	69.01	76.35	84.26	93.65
18.5	55.42	59.46	64.14	70.22	77.47	85.30	94.61

**Table 7:** Percentile values and z-score of body weight in girls derived from the nationwide anthropometric survey 2001 in Slovakia

z-score	-1.881	-1.282	-0.674	0	0.674	1.282	1.881
Percentile	3	10	25	50	75	90	97
Age (years)							
7.0	17.54	19.03	20.80	23.17	26.09	29.38	33.47
7.5	18.34	19.94	21.85	24.40	27.57	31.14	35.61
8.0	19.20	20.93	22.99	25.75	29.19	33.08	37.96
8.5	20.11	21.97	24.20	27.20	30.94	35.18	40.51
9.0	21.07	23.09	25.50	28.76	32.83	37.46	43.27
9.5	22.10	24.29	26.92	30.47	34.90	39.93	46.26
10.0	23.21	25.60	28.47	32.34	37.18	42.65	49.50
10.5	24.45	27.06	30.20	34.42	39.68	45.60	52.97
11.0	25.85	28.70	32.12	36.71	42.39	48.74	56.57
11.5	27.41	30.50	34.19	39.12	45.17	51.88	60.06
12.0	29.16	32.46	36.39	41.59	47.93	54.88	63.26
12.5	31.09	34.56	38.65	44.04	50.55	57.62	66.04
13.0	33.17	36.75	40.93	46.42	52.98	60.05	68.39
13.5	35.31	38.92	43.14	48.63	55.15	62.12	70.30
14.0	37.34	40.95	45.14	50.57	56.97	63.79	71.73
14.5	39.16	42.74	46.87	52.19	58.46	65.08	72.77
15.0	40.71	44.24	48.31	53.53	59.64	66.08	73.52
15.5	41.97	45.45	49.45	54.57	60.54	66.82	74.05
16.0	42.93	46.37	50.32	55.35	61.21	67.35	74.41
16.5	43.64	47.05	50.95	55.92	61.69	67.72	74.65
17.0	44.16	47.54	51.41	56.32	62.03	67.99	74.81
17.5	44.55	47.92	51.76	56.64	62.29	68.19	74.93
18.0	44.89	48.24	52.06	56.90	62.51	68.36	75.04
18.5	45.22	48.55	52.34	57.15	62.72	68.51	75.13

**Table 8:** Percentile values and z-score of body mass index in boys derived from the nationwide anthropometric survey 2001 in Slovakia

z-score	-1.881	-1.282	-0.674	0	0.674	1.282	1.881
Percentile	3	10	25	50	75	90	97
Age (years)							
7.0	12.83	13.53	14.37	15.49	16.91	18.55	20.69
7.5	12.88	13.58	14.42	15.56	17.01	18.72	21.01
8.0	12.95	13.66	14.51	15.67	17.16	18.94	21.39
8.5	13.05	13.76	14.63	15.81	17.34	19.21	21.82
9.0	13.17	13.89	14.76	15.97	17.55	19.50	22.29
9.5	13.29	14.03	14.92	16.15	17.79	19.82	22.77
10.0	13.43	14.18	15.09	16.35	18.03	20.14	23.25
10.5	13.58	14.34	15.27	16.57	18.30	20.47	23.71
11.0	13.75	14.53	15.48	16.80	18.58	20.81	24.15
11.5	13.93	14.73	15.70	17.06	18.87	21.15	24.55
12.0	14.14	14.95	15.95	17.34	19.19	21.51	24.93
12.5	14.36	15.20	16.22	17.64	19.52	21.87	25.30
13.0	14.61	15.47	16.51	17.96	19.87	22.23	25.64
13.5	14.88	15.76	16.83	18.30	20.24	22.61	25.97
14.0	15.16	16.06	17.16	18.66	20.61	22.98	26.29
14.5	15.45	16.38	17.49	19.02	20.98	23.34	26.59
15.0	15.74	16.69	17.83	19.37	21.35	23.70	26.87
15.5	16.04	17.00	18.16	19.72	21.71	24.05	27.15
16.0	16.33	17.31	18.49	20.07	22.06	24.38	27.42
16.5	16.61	17.61	18.80	20.40	22.40	24.70	27.68
17.0	16.88	17.91	19.12	20.73	22.73	25.02	27.93
17.5	17.15	18.19	19.42	21.05	23.05	25.32	28.18
18.0	17.42	18.48	19.72	21.36	23.37	25.62	28.43
18.5	17.68	18.76	20.01	21.67	23.68	25.92	28.68

**Table 9:** Percentile values and z-score of body mass index in girls derived from the nationwide anthropometric survey 2001 in Slovakia

z-score	-1.881	-1.282	-0.674	0	0.674	1.282	1.881
Percentile	3	10	25	50	75	90	97
Age (years)							
7.0	12.49	13.26	14.16	15.34	16.77	18.34	20.26
7.5	12.54	13.32	14.23	15.44	16.91	18.54	20.56
8.0	12.61	13.41	14.34	15.58	17.10	18.81	20.94
8.5	12.71	13.52	14.48	15.75	17.33	19.12	21.38
9.0	12.83	13.66	14.64	15.96	17.60	19.47	21.86
9.5	12.97	13.82	14.83	16.19	17.89	19.85	22.37
10.0	13.12	14.00	15.04	16.45	18.21	20.25	22.90
10.5	13.29	14.19	15.27	16.72	18.56	20.67	23.42
11.0	13.49	14.42	15.53	17.02	18.91	21.10	23.94
11.5	13.71	14.66	15.80	17.34	19.28	21.52	24.42
12.0	13.95	14.93	16.10	17.67	19.65	21.93	24.87
12.5	14.22	15.22	16.41	18.01	20.02	22.32	25.27
13.0	14.51	15.53	16.74	18.36	20.38	22.68	25.63
13.5	14.81	15.84	17.06	18.69	20.72	23.02	25.94
14.0	15.11	16.15	17.38	19.02	21.04	23.33	26.21
14.5	15.41	16.45	17.68	19.32	21.33	23.59	26.42
15.0	15.69	16.73	17.96	19.59	21.58	23.82	26.60
15.5	15.96	17.00	18.22	19.83	21.81	24.01	26.75
16.0	16.20	17.23	18.45	20.05	22.00	24.17	26.86
16.5	16.42	17.45	18.65	20.23	22.16	24.30	26.95
17.0	16.63	17.64	18.83	20.40	22.30	24.41	27.01
17.5	16.82	17.83	19.00	20.55	22.43	24.50	27.06
18.0	17.01	18.00	19.17	20.69	22.54	24.59	27.10
18.5	13.12	13.97	19.33	20.84	22.66	24.67	27.14

Median BMI in 2001 was almost identical to WHO 2007 standards, except in 16-18-year-old girls, with z-score -0.25 or 40th percentile (Figure 1B). Median BMI in 2011 in the age group of 9-15 years significantly increased by 0.7-1.6 IU (*P* < 0.001). However, in older girls it returned to zero, whereas in boys it remained higher by 0.5 IU than Slovak and WHO 2007 standards. The respective z-scores according to WHO 2007 standards in boys ranged from 0.2 to 0.7 IU, and in girls up to 0.5 IU (Figure 1).

### Overweight and obesity definitions

**Figure 2:**
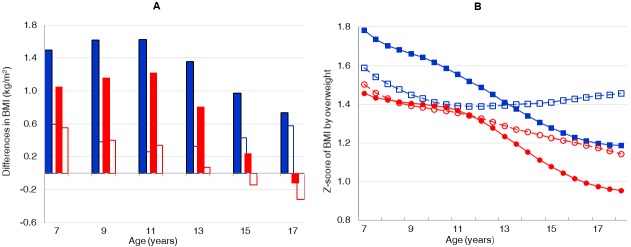
Comparison of Slovak and World Health Organization (WHO) 2007 or International Obesity Task Force (IOTF) cut-offs for overweight expressed as differences in body mass index (BMI) values; blue – boys by WHO, red – girls by WHO; empty, blue border – boys by IOTF, empty, red border – girls by IOTF (**A**) and respective z-scores; filled square – boys by WHO, filled circle – girls by WHO, open square – boys by IOTF, open circle – girls by IOTF (**B**).

The cut-offs for overweight and obesity in Slovakia were traditionally set at the 90th and 97th percentile of BMI, respectively. We compared these cut-offs with WHO 2007 and IOTF definitions expressed as z-scores and IU of BMI (Figure 2). The cut-offs for overweight in boys were higher (0.6-1.7 IU) compared with both WHO 2007 and IOTF standards, corresponding to z-scores of 1.2-1.8. In girls, the differences did not exceed 1.2 IU or z-score 1.5. Discrepancies with WHO 2007 standards in both sexes decreased with age. Discrepancies with IOTF standards ranged from -0.3 IU (17-year-old girls) to 1.3 IU (7-year-old boys).

**Figure 3:**
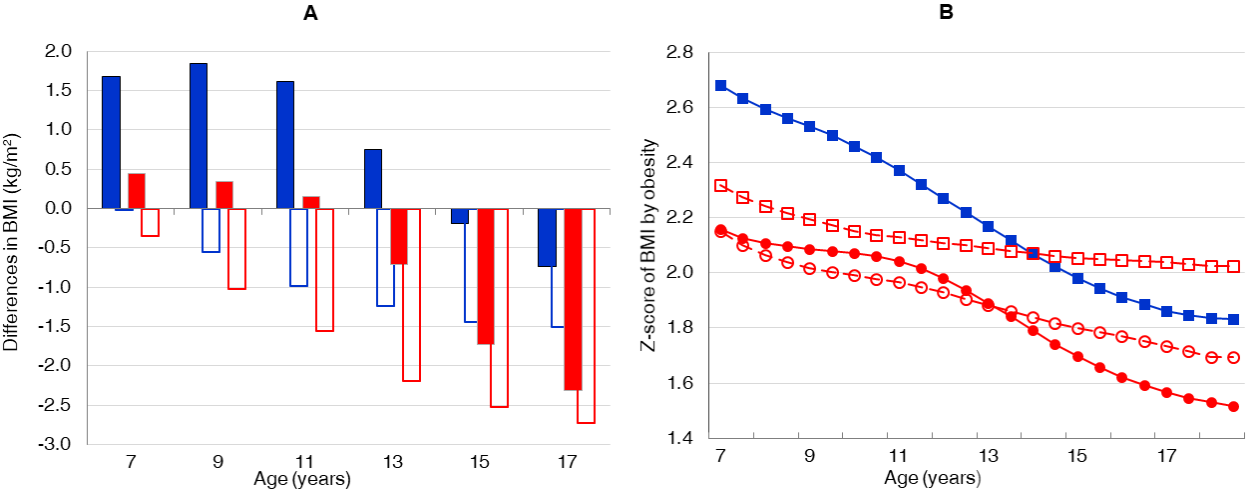
Comparison of Slovak and World Health Organization (WHO) 2007 or International Obesity Task Force (IOTF) cut-offs for obesity expressed as differences in body mass index (BMI) values; blue – boys vs WHO, red – girls vs WHO; empty, blue border – boys vs IOTF, empty, red border – girls vs IOTF (**A**) and respective z-scores; filled square – boys by WHO, filled circle – girls by WHO, open square – boys by IOTF, open circle – girls by IOTF (**B**).

The cut-offs for obesity in younger boys matched IOTF definitions. WHO 2007 cut-offs for boys up to 13 years of age were much lower (by 1.0-1.9 IU) than Slovak and IOTF cut-offs. These disparities decreased with age, and the values from three classifications became similar, corresponding to z-scores of 1.8-2.2 (Figure 3). Slovak cut-offs for younger girls were similar to WHO 2007 cut-offs, but cut-offs for older girls were lower by 1.5-2.5 IU than WHO 2007 and by 2.0-3.0 IU than IOTF limits (z-score 1.5-1.8) (Figure 3).

### Obesity prevalence

**Figure 4:**
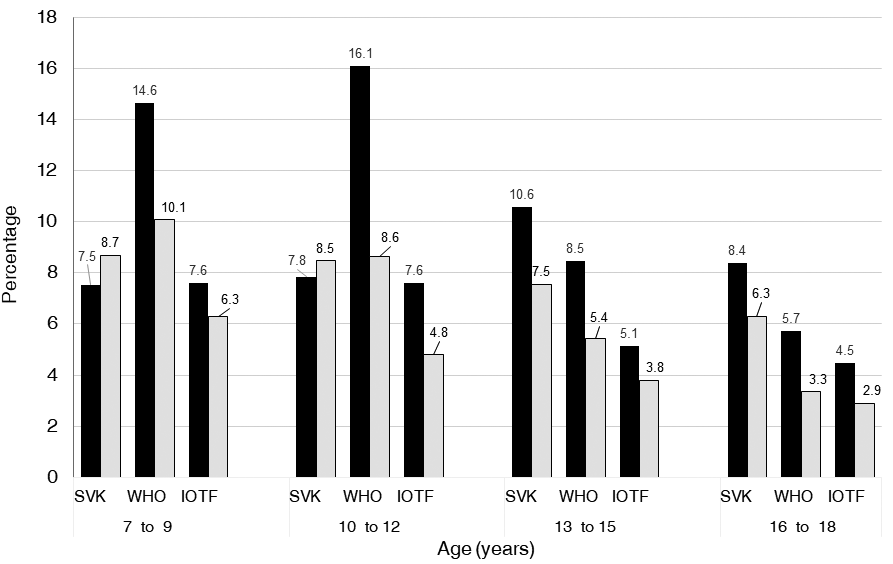
Prevalence of obesity in 2011 using Slovak (SVK), World Health Organization (WHO) 2007, and International Obesity Task Force (IOTF) standards. Black – boys; gray – girls.

WHO 2007 obesity cut-offs for boys are set at lower BMI values (by 0.6-0.8 IU) than those for girls. Consequently, obesity prevalence in age groups <13 years was highest when WHO 2007 cut-offs were used (Figure 4). When Slovak and IOTF cut-offs were used, it yielded similar results in boys, while in girls it was lowest when IOTF standards were used. In children aged up to 11 years, no sex differences were found when either of these cut-offs was used. However, obesity rates according to WHO 2007 standards in boys aged up to 13 years were nearly two times higher than in girls and much higher than IOTF or Slovak standards. In older age groups, they were highest when Slovak standards were used. Obesity prevalence in girls decreased with age, regardless of the standards used.

**Figure 5:**
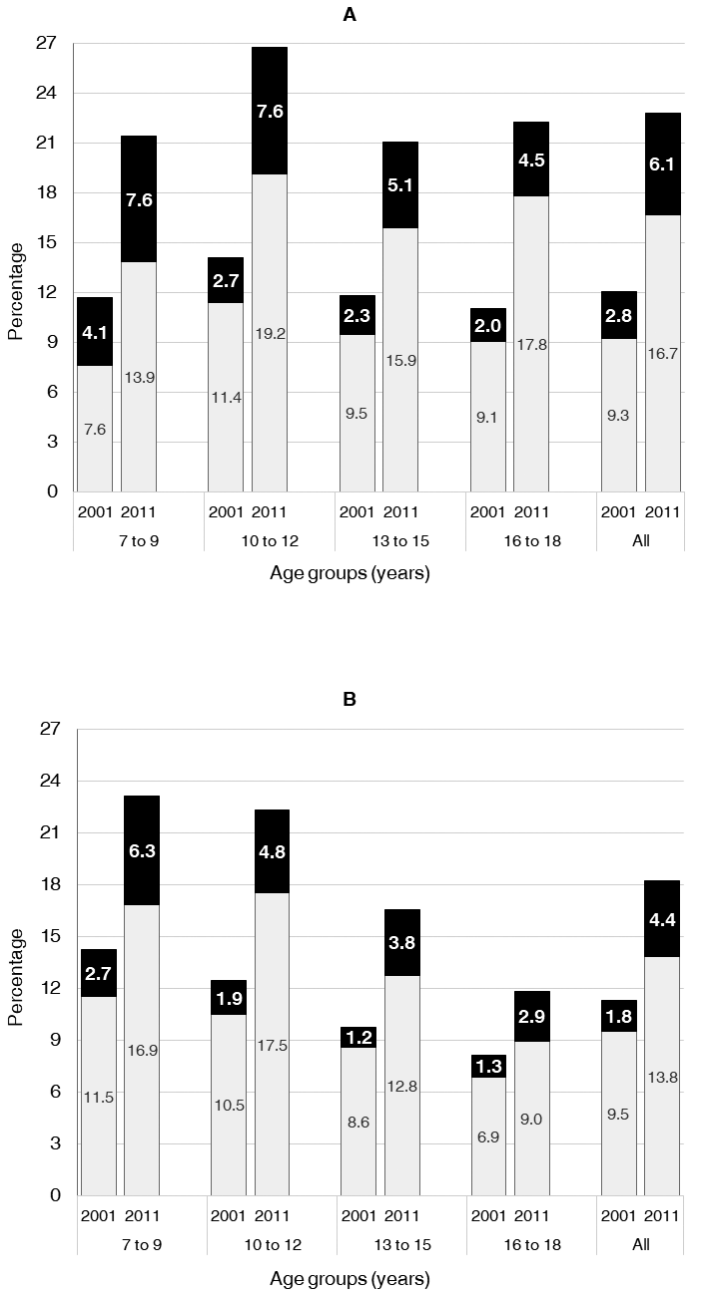
Prevalence of overweight and obesity according to International Obesity Task Force (IOTF) limits in 2001 and 2011 in (**A**) boys and (**B**) girls. All differences between the proportions in 2001 and 2011 were significant at *P* < 0.001, but the level of significance for overweight in girls 16–18 years was *P* < 0.005. Open – overweight; filled – obesity.

As Slovak standards (set at 90th and 97th percentile) were derived from NAS 2001 data, the overweight and obesity rates that year nearly met the theoretically expected values of 7% and 3%, respectively. In the following 10 years, these proportions doubled. For cross-country comparisons, we chose IOTF cut-offs (Figure 5) to evaluate the changes during the 2001-2011 period. In NAS 2011, overweight prevalence was higher (*P* < 0.001) in all subgroups, but the trend rise for obesity was even steeper, as it more than doubled across all ages. The highest increase in overweight, including obesity, in girls was observed in the age group 10-12 years (by 10%), although in the oldest age group it was only 3.7%. In boys, the prevalence was higher than in girls (*P* < 0.001), but its age-related increment variations were lower (10%-13%) (Figure 5).

## Discussion

We confirmed our hypothesis that higher weight increments in NAS 2011 were not proportional to growth, which supports the choice of reference values based on the NAS 2001 data.

### Overweight and obesity definitions

Sex- and age-specific BMI cut-offs based on NAS 2001 were lower than other nation-specific and IOTF standards. Cut-offs derived from 2011 data may be modified by an increase in obesity (14,15,23-25) and are relatively high compared with Slovak or Czech references (12). Although our hypothesis about differences between nation-specific reference values and international standards was confirmed, we did not expect such great discrepancies in sex and age, mainly from WHO 2007 cut-offs. These discrepancies cannot be explained only by body height variations and secular trends in respective age groups and the entire population. We have recently pointed out these inconsistencies in a study on 2795 children aged 7 years under the WHO European Childhood Obesity Surveillance Initiative (COSI) project in Slovakia (26). The obesity prevalence in boys in Slovakia and in other countries participating in the COSI study (27) was higher compared to girls only using WHO 2007 standards. Such differences were not demonstrated when IOTF or nation specific references were used. In 8 out of 13 countries, obesity prevalence according to IOTF standards was even higher in girls. The present study showed that this also applied to older age groups.

WHO 2007 standards are derived from data recorded about 40 years ago, so they may have become outdated due to secular trends and growth acceleration. The contemporary school-age children in industrial Europe are by 3-6 cm taller than WHO standards (10,28-32), which was also documented by consistently positive mean height z-scores. Taller children systematically showed higher BMI values, while shorter children showed lower median BMI values (30,32).

According to WHO 2007 definitions of overweight and obesity (1), boys under 13 years have BMI lower by 0.8 IU than girls of the same age. For example, a 9.5-year-old boy with the weight of 38.1 kg and height of 135 cm would be classified as obese, but a girl of the same age and height weighing 40 kg would be classified as overweight. Likewise, a girl weighing 34 kg would be classified as normal, but a boy weighing 33.2 kg would be classified as overweight. According to the WHO classification, the portion of boys up to the age of 13 years who were identified as obese was implausibly higher compared with girls of the same age. According to Slovak national cut-offs, 12 228 boys and 13 724 girls were considered obese, while according to WHO cut-offs, 24 368 boys (two times more) and 14 841 girls (an almost equal number) were considered obese. Such great sex differences were not observed when obesity was assessed according to IOTF.

Since girls aged 11-12 years are only slightly taller and heavier than boys (12,14,15,28,30,31), the question arises if there is a need for stricter BMI limits for prepubertal boys. In both 2001 and 2011 surveys, the prevalence of overweight, including obesity, in girls started to differ approximately at the age of 13-14 years and lowered with age. Girls start quite early with an effort to remain slender and are at risk of harming their health with inappropriate diet.

In contrast to IOTF standards (3), Slovak BMI-age curves do not pass through the BMI of 30 for obesity at the age of 18, as the 97th percentile represents much lower values, especially in girls. Therefore, they may overestimate the obesity prevalence in older age groups. On the other hand, Slovak BMI references match IOTF standards for children up to 10 years, and so they yield similar obesity rates. When applying IOTF criteria, our results are in good agreement with the recent Organisation for Economic Co-operation and Development statistics, which show a relatively low prevalence of obesity in Slovaks younger than 20 years (16).

### Obesity prevalence

Previous nationwide anthropometric surveys (4,7-10) have shown secular trends in all anthropometric parameters, mainly in body height. Toward the end of the 20th century, the population was getting slimmer (7). In our study, prepubertal and pubertal age groups grew slightly during the first decade of the new millennium, but the final body height (of the oldest age group) remained the same. Slovak girls reached their “adult” body height at 16 years, which is a trend first revealed in 1991 (7,9). At the same time, intensive weight gain was observed, especially among boys, which markedly increased the overweight and obesity proportions. Although overweight and obesity prevalence remain among the lowest in Central and Western European countries (16), the steep obesity rise is worrisome.

Regardless of its limitations as a measure of adiposity in children and adolescents (33-37), BMI is widely accepted as the simplest tool to unveil global time population trends and detect developmental deviations on an individual level. These goals can best be achieved using nation-specific reference values for the assessment of growth and proportionality of physical characteristics.

In Slovak children, the “obesity epidemics” started approximately at the beginning of the new millennium, which is 15-20 years later than in other industrial and economically developed European countries. However, this initially slightly rising trend became more abrupt during the period 2001-2011, regardless of the references used. When obesity started to rise in Slovakia, it plateaued or declined in many other European countries (38-43). The NAS in 2021 will show whether Slovakia will face a similar trend. However, the most recent research (26) has shown the culmination of obesity rise approximately 6 years ago.

The strengths of the current study are uniform internationally accepted methodology and the nationally representative samples obtained in both surveys, with relatively high participation rates proportional to the population size in all parts of Slovakia (21). However, the study also has some limitations. Both surveys recruited voluntary participants, meaning that adolescents, especially girls, who refused to be measured might have been more obese. This could affect not only the overall obesity prevalence, which was lower in adolescents than in prepubertal children, but also the cut-off BMI values, which were lower than in most of the European countries in girls older than 16 years. Additionally, compulsory education in Slovakia ends at the age of 16, so the surveys did not include older adolescents who did not continue their education after that age.

The comparison of long-term obesity prevalence trends showed that although obesity prevalence doubled since the NAS 2001, it is still lower than in the majority of European countries. Obesity definition according to WHO 2007, set at much lower BMI values in boys than in girls, may overestimate the prevalence in boys. The revealed differences in the growth rate of Slovak children and adolescents justify the creation of a national reference of anthropometric data.

## References

[1] de Onis M, Onyango AW, Borghi E, Siyam A, Nishida C, Siekmann J (2007). Development of a WHO growth reference for school-aged children and adolescents.. Bull World Health Organ.

[2] Kuczmarski RJ, Ogden CL, Guo SS, Grummer-Strawn LM, Flegal KM, Mei Z (2002). 2000 CDC Growth Charts for the United States: methods and development.. Vital Health Stat 11.

[3] Cole TJ, Lobstein T (2012). Extended international (IOTF) body mass index cut-offs for thinness, overweight and obesity: Extended international BMI cut-offs.. Pediatr Obes.

[4] Lipková V, Grunt J (1984). Physical development of children and youth in Slovakia in the recent period. Čs Hyg.

[5] Prokopec M, Zlámalová H, Lipková V, Grunt J (1991). Comparison of basic somatic dimensions of Czech and Slovak children and youth. Cesk Pediatr.

[6] Prokopec M, Lhotská L, Tomásek L, Vígnerová J (1991). The effect of heredity and environment on growth, body weight and physical proportions in children and adolescents. Cesk Pediatr.

[7] Nováková J, Ševčíková Ľ (1994). Changes of somatometric indicators of Slovak children and youth in 1991 as compared with 1981. Hygiena..

[8] Ševčíková Ľ, Jurkovičová J, Štefániková Z, Macháčová E, Sobotová Ľ, Ághová Ľ (2006). The environmental conditions, health, and development of children and youth.. Homeostasis..

[9] Ševčíková Ľ, Rovný I, Nováková J, Hamade J, Tatara M, Janechová H, et al. Physical development of children and adolescents in Slovakia Results of the VI^th^ National survey in the year of 2001. [Telesný vývoj detí a mládeže v SR]. Bratislava: Public Health Authority of the Slovak Republic; 2004.

[10] Kovács L, Babinská K, Ševčíková Ľ, Hamade J, Jurkovičová J, Štefániková Z, et al. New trends in nourishment in children. [Nové trendy vo výžive detí]. Bratislava: Comenius University; 2007.

[11] Cole TJ (2000). Establishing a standard definition for child overweight and obesity worldwide: international survey.. BMJ.

[12] Vignerová J, Humeníkova L, Brabec M, Riedlová J, Bláha P (2007). Long-term changes in body weight, BMI, and adiposity rebound among children and adolescents in the Czech Republic.. Econ Hum Biol.

[13] Eiben OG, Tóth GA, VanWieringen JC. Weight/height indices in Hungarian youth during the twentieth century. In: Singh SP, Gaur R, editors. Human body composition. Delhi: Kamla-Raj Enterprise; 2007. p. 9-16.

[14] Mayer M, Gleiss A, Häusler G, Borkenstein M, Kapelari K, Köstl G (2015). Weight and body mass index (BMI): current data for Austrian boys and girls aged 4 to under 19 years.. Ann Hum Biol.

[15] Kułaga Z, Litwin M, Tkaczyk M, Palczewska I, Zajączkowska M, Zwolińska D (2011). Polish 2010 growth references for school-aged children and adolescents.. Eur J Pediatr.

[16] Ng M, Fleming T, Robinson M, Thomson B, Graetz N, Margono C (2014). Global, regional, and national prevalence of overweight and obesity in children and adults during 1980-2013: a systematic analysis for the Global Burden of Disease Study 2013.. Lancet.

[17] (2017). Worldwide trends in body-mass index, underweight, overweight, and obesity from 1975 to 2016: a pooled analysis of 2416 population-based measurement studies in 128·9 million children, adolescents, and adults.. Lancet.

[18] Tanner JM, Hiernaux J, Jarman S. Growth and physique studies. In: Weiner JS, Lourie JA, editors. Human biology. A guide to field methods. IBP Handbook No. 9. Oxford, UK: Blackwell Scientific Publications; 1969: p. 1-42.

[19] WHO Expert Committee on Physical Status. The use and interpretation of anthropometry: a report of a WHO Expert Committee. Geneva: World Health Organization; 1995. p. 452. 8594834

[20] Pan H, Cole TJ. LMSchartmakerPro, a program to construct growth references using the LMS method Version 2.54 [software]. Medical Research Council the Institute of Child Health, London. c 1997-2011. Available from: http://www.healthforallchildren.com/shop-base/shop/software/lmschartmaker-pro//*.* Accessed: April 25, 2011.

[21] Cole TJ, Green PJ (1992). Smoothing reference centile curves: The lms method and penalized likelihood.. Stat Med.

[22] Pan H, Cole TJ. LMSgrowth, a Microsoft Excel add-in to access growth references based on the LMS method. 2.77 ed. [software]. Medical Research Council the Institute of Child Health, London, 2002-2012. Available from*:* http://www.healthforallchildren.com/lmsgrowth-download//*.* Accessed: December 12, 2012.

[23] Rosario AS, Kurth B-M, Stolzenberg H, Ellert U, Neuhauser H (2010). Body mass index percentiles for children and adolescents in Germany based on a nationally representative sample (KiGGS 2003-2006).. Eur J Clin Nutr.

[24] Pérez-Farinós N, López-Sobaler AM, Ángels Dal Re M, Villar C, Labrado E, Robledo T (2013). The ALADINO Study: A National Study of Prevalence of Overweight and Obesity in Spanish Children in 2011.. BioMed Res Int.

[25] Brettschneider A-K, Schienkiewitz A, Schmidt S, Ellert U, Kurth B-M (2017). Updated prevalence rates of overweight and obesity in 4- to 10-year-old children in Germany. Results from the telephone-based KiGGS Wave 1 after correction for bias in parental reports.. Eur J Pediatr.

[26] Tichá Ľ, Regecová V, Šebeková K, Sedláková D, Hamade J, Podracká Ľ (2018). Prevalence of overweight/obesity among 7-year-old children-WHO Childhood Obesity Surveillance Initiative in Slovakia, trends and differences between selected European countries.. Eur J Pediatr.

[27] Wijnhoven TMA, van Raaij JM, Spinelli A, Starc G, Hassapidou M, Spiroski I (2014). WHO European Childhood Obesity Surveillance Initiative: body mass index and level of overweight among 6-9-year-old children from school year 2007/2008 to school year 2009/2010.. BMC Public Health.

[28] Cacciari E, Milani S, Balsamo A, Dammacco F, De Luca F, Chiarelli F (2002). Italian cross-sectional growth charts for height, weight and BMI (6-20 y).. Eur J Clin Nutr.

[29] Haas JD, Campirano F (2006). Interpopulation variation in height among children 7 to 18 years of age.. Food Nutr Bull.

[30] Rosario AS, Schienkiewitz A, Neuhauser H (2011). German height references for children aged 0 to under 18 years compared to WHO and CDC growth charts.. Ann Hum Biol.

[31] Starc G, Strel J (2011). Is there a rationale for establishing Slovenian body mass index references of school-aged children and adolescents?. Anthropol Noteb.

[32] Bonthuis M, van Stralen KJ, Verrina E, Edefonti A, Molchanova EA, Hokken-Koelega ACS (2012). Use of national and international growth charts for studying height in European children: development of up-to-date European height-for-age charts.. PLoS One.

[33] Keys A, Fidanza F, Karvonen MJ, Kimura N, Taylor HL (2014). Indices of relative weight and obesity.. Int J Epidemiol.

[34] Wellens RI, Roche AF, Khamis HJ, Jackson AS, Pollock ML, Siervogel RM (1996). Relationships between the body mass index and body composition.. Obes Res.

[35] Siervogel RM, Maynard LM, Wisemandle WA, Roche AF, Guo SS, Chumlea WC (2000). Annual changes in total body fat and fat-free mass in children from 8 to 18 years in relation to changes in body mass index. The Fels Longitudinal Study.. Ann N Y Acad Sci.

[36] Neovius MG, Linné YM, Barkeling BS, Rossner SO (2004). Sensitivity and specificity of classification systems for fatness in adolescents.. Am J Clin Nutr.

[37] Nuttall FQ (2015). Body Mass Index: obesity, BMI, and health.. Nutr Today.

[38] Mitchell RT, McDougall CM, Crum JE (2007). Decreasing prevalence of obesity in primary schoolchildren.. Arch Dis Child.

[39] Olds T, Maher C, Zumin S, Péneau S, Lioret S, Castetbon K (2011). Evidence that the prevalence of childhood overweight is plateauing: data from nine countries.. Int J Pediatr Obes.

[40] Blüher S, Meigen C, Gausche R, Keller E, Pfäffle R, Sabin M (2011). Age-specific stabilization in obesity prevalence in German children: A cross-sectional study from 1999 to 2008.. Int J Pediatr Obes.

[41] Moss A, Klenk J, Simon K, Thaiss H, Reinehr T, Wabitsch M (2012). Declining prevalence rates for overweight and obesity in German children starting school.. Eur J Pediatr.

[42] Schmidt Morgen C, Rokholm B, Sjöberg Brixval C, Schou Andersen C, Geisler Andersen L, Rasmussen M (2013). Trends in prevalence of overweight and obesity in Danish infants, children and adolescents – are we still on a plateau?. PLoS One.

[43] Wabitsch M, Moss A, Kromeyer-Hauschild K (2014). Unexpected plateauing of childhood obesity rates rates in developed countries.. BMC Med.

